# Conformational Heterogeneity of Bax Helix 9 Dimer for Apoptotic Pore Formation

**DOI:** 10.1038/srep29502

**Published:** 2016-07-06

**Authors:** Chenyi Liao, Zhi Zhang, Justin Kale, David W. Andrews, Jialing Lin, Jianing Li

**Affiliations:** 1 Department of Chemistry, University of Vermont, Burlington, VT 05405, USA; 2 Department of Biochemistry and Molecular Biology, University of Oklahoma Health Sciences Center, Oklahoma City, OK 73126, USA; 3 Biological Sciences, Sunnybrook Research Institute, University of Toronto, Toronto, ON, Canada; 4 Department of Biochemistry, McMaster University, Hamilton, ON, Canada; 5 Departments of Biochemistry and Medical Biophysics, University of Toronto, Toronto, ON, Canada; 6 Stephenson Cancer Center, Oklahoma City, OK 73104, USA

## Abstract

Helix α9 of Bax protein can dimerize in the mitochondrial outer membrane (MOM) and lead to apoptotic pores. However, it remains unclear how different conformations of the dimer contribute to the pore formation on the molecular level. Thus we have investigated various conformational states of the α9 dimer in a MOM model — using computer simulations supplemented with site-specific mutagenesis and crosslinking of the α9 helices. Our data not only confirmed the critical membrane environment for the α9 stability and dimerization, but also revealed the distinct lipid-binding preference of the dimer in different conformational states. In our proposed pathway, a crucial iso-parallel dimer that mediates the conformational transition was discovered computationally and validated experimentally. The corroborating evidence from simulations and experiments suggests that, helix α9 assists Bax activation via the dimer heterogeneity and interactions with specific MOM lipids, which eventually facilitate proteolipidic pore formation in apoptosis regulation.

As a proapoptotic protein from the Bcl-2 family, Bax is a crucial executioner in the mitochondrial pathway of apoptosis^1^,^2^,^3^. In stressed cells, Bax from the cytosol is activated by the BH3-only proteins (such as Bim and Bid) and inserts into the mitochondrial outer membrane (MOM)^4^,^5^,^6^,^7^,^8^,^9^. Through multi-step conformational changes, Bax forms dimers and further oligomerizes into membrane permeabilizing pores^8^,^9^,^10^,^11^ which subsequently release apoptotic mitochondrial proteins like cytochrome c and SMAC to the cytosol and lead to cell death. Nonetheless, the conformational transition of Bax after activation, involving the dimerization in the cytosolic region and/or the MOM, has not been fully understood. Upon activation, a symmetric dimer conformation with reciprocal binding of the BH3 region of one Bax to the groove of another Bax was first observed by X-ray crystallography using a truncated Bax protein^8^, and recently confirmed by crosslinking of intact Bax protein in the mitochondria^12^. While Bax dimerization has been confirmed in certain cytosolic domains, the MOM-anchoring domain — in particular, the helix α9 (residue 169–189) — is found to form dimer interfaces that expand the oligomeric pore in the MOM^10^,^12^,^13^. In the soluble Bax, helix α9 is buried in a hydrophobic groove formed by helices α2, α3, α4, and α5^11^. However, studies using Förster resonance energy transfer (FRET) and electron spin resonance (ESR) observed significant rearrangements of helix α9 indicating its exposure to the cytosol before membrane binding^10^,^14^. In the activated Bax, helix α9 functions as a membrane anchor to target Bax to the MOM, because its deletion could prevent Bax translocation to the mitochondria^15^. Consistently, mutations of Ser184 in helix α9 could either abolish or enhance the Bax translocation^15^. While prior studies pointed out that helix α9 would be a key to understand Bax activation and apoptosis regulation, its particular roles and relevant molecular mechanisms have not been fully understood.

Recently we have discovered two distinct conformational states of the α9 dimer in the MOM. The first state, termed the *intersected* α9 dimer (Fig. 1A1), was based on the disulfide linkages of four cysteine mutants: I175C, G179C, A183C and I187C^12^. In this state, two α9 helices intersect though the G^179^xxxA^183^ motif with a right-handed crossing angle of ~40°^16^. The other state is referred to as the *parallel* α9 dimer (Fig. 1B1) with a right-handed crossing angle below 15°, according to the disulfide linkages of other four cysteine mutants: Q171C, A178C, T182C and L185C^12^. Although α9 dimerization was believed to facilitate the lipidic pore^12^, the actual roles of these two dimeric states and their connections to Bax activation remain elusive or controversial. A number of key problems on the molecular level need to be studied. First, despite other mitochondrial targeting signals in Bax, helix α9 by itself is sufficient to target the mitochondria^17^. However, what drives helix α9 into the MOM, and how is helix α9 stabilized in the membrane is currently unknown. Moreover, mitochondrial specific lipids are known to actively influence the structures and functions of the Bcl-2 family proteins including Bax^18^. It has been suggested that insertion of helix α9 into the MOM and Bax pore formation are regulated by the membrane curvature and lipid compositions^19^. Then how does helix α9 interact with various lipids in the MOM, especially the mitochondrion-specific ones? What is the significance of these interactions for helix α9 dimerization and mitochondrial poration? In an attempt to gain molecular insight, we herein present the first systematic study of molecular dynamics (MD) simulations for the helix α9 dimer in a model lipid bilayer, in tandem with experimental investigations of the helix α9 dimer interface between intact Bax proteins activated in the native MOM.

While MD simulations have been applied to study the stability of helix α9 bound to the Bax groove in the aqueous solution^20^,^21^,^22^, a large-scale simulation study of helix α9 with atomic details in the membrane has never been reported. Also, it is required to further investigate the roles of Bax helix α9 in apoptosis with a detailed membrane model to mimic the MOM lipid composition. Therefore, in this work we focus on the helix α9 dimers in a MOM-mimicking lipid bilayer, using conventional all-atom MD and free-energy simulations for both qualitative and quantitative analyses. Simulations starting from the “static” intersected and parallel dimer models^12^ were performed to understand the conformational dynamics, while in return, experiments with full-length Bax variants in native mitochondria were designed to validate the predictions from the simulations. Such synergy between simulations and experiments enable us to access structural and dynamic details of helix α9 at various spatial and temporal scales. Building on good agreements between simulations and experiments, our work shines light on the molecular mechanism of Bax dimerization via helix α9 interactions in the MOM, which has never been fully described before. In addition, our study establishes a unique approach for further investigations of the apoptotic MOM permeabilization induced by Bax and regulated by other Bcl-2 family proteins.

## Results

### The membrane environment is critical to stabilize the helix α9 dimers

**Figure 1:**
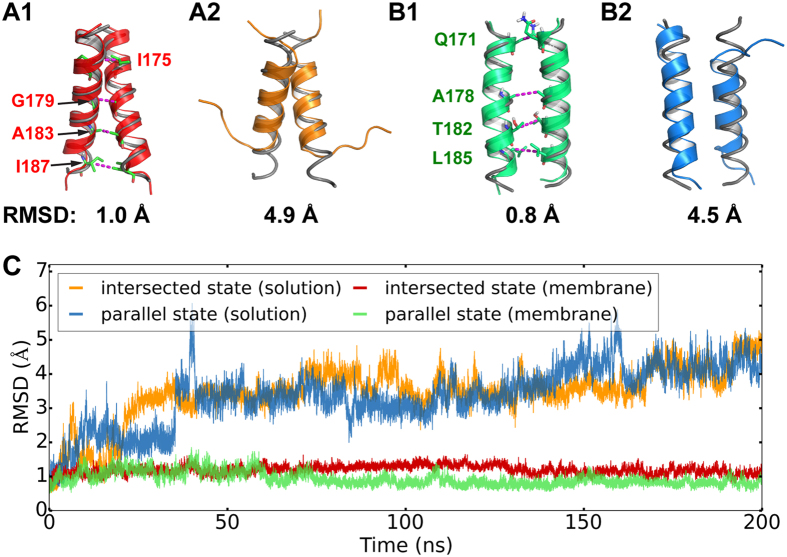
Comparison of the intersected (**A**) and parallel (**B**) helix α9 dimers in the membrane (**A1,B1**) and solution (**A2,B2**) environments. The final dimer conformations in the cartoon representation are superimposed onto the starting ones as grey tubes, with the RMSDs indicated below. The residue pairs that could be crosslinked by disulfides after replaced by cysteine pairs are highlighted with the stick representations linked by dashed lines. (**C**) Plots of the dimer backbone RMSDs over time, with the starting conformations (*t* = 0 ns) as the references. The second replicas reproduced the qualitative results (Supplementary Fig. 2).

We have compared the stabilities of the intersected and parallel α9 dimers in a MOM-mimicking lipid bilayer versus an aqueous solution. Within the 200-ns MD simulations, both the intersected and parallel α9 dimers appear stable in the membrane (Fig. 1), as shown by the small fluctuation around 1 Å of the backbone root-mean-square deviation (RMSD). Consistently, all the cross-linkable data^12^ were reproduced by C_β_-C_β_’ distances along time evolution (Supplementary Fig. 1). In contrast, in solution the α9 dimers became disordered during the simulations (A2 and B2 of Fig. 1). Compared to the starting conformations, the backbone RMSDs of both dimers in the solution dramatically increase in the first 50 ns and then fluctuate around 5 Å toward the end of the simulations (Fig. 1C). The helicity of the intersected dimer decreases from 87 to 54% due to melting in the terminal regions, giving rise (see Supplementary Fig. 3) to a separation greater than 27 Å for I187-I187′ pair in the C-termini. Similarly, the helicity of the parallel dimer drops from 87 to 70%, increasing Q171-Q171′ and T182-T182′ separations to 13 and 11 Å, respectively. Additionally, rotation of the individual α9 helices in the parallel dimer was also observed in the solution, which increased the crossing angle between two monomers by over 10°. Likewise for a single α9 monomer in the solution, a dramatic loss of helicity was observed in our simulations.

Our MD simulations demonstrate that the membrane environment is essential to maintain the helicity of α9, because α9 remains helical in the membrane but becomes disordered in the solution. Combined with the previous experimental finding of helix α9 being partially buried in a groove of the solvated Bax monomer^11^, we suggest a possible conformational transition of helix α9 after Bax activation upon binding to a BH3 peptide or protein that displaces α9 from the groove^5^,^8^. It is likely that an order-to-disorder transition occurs after helix α9 becomes unbound to the groove of Bax, and a disorder-to-order transition follows when helix α9 starts to insert into the MOM. α9 adopts disordered conformations due to the exposure of its hydrophobic core to the cytosol or even membrane’s charged periphery, while the membrane plays a key role in recovering the peptide helicity by providing a hydrophobic environment around α9’s hydrophobic core and charged/polar periphery around α9’s charged/polar termini. In fact, many peptides such as melittin^23^,^24^ are known to undergo similar disorder-to-order transitions when inserted to membranes. Therefore, the order (in groove)-to-disorder (in cytosol)-to-order (in membrane) transition may be a general pathway for α9 targeting and insertion to the MOM during Bax activation.

#### The intersected and parallel α9 dimers display distinct preferences toward anionic lipids

**Figure 2:**
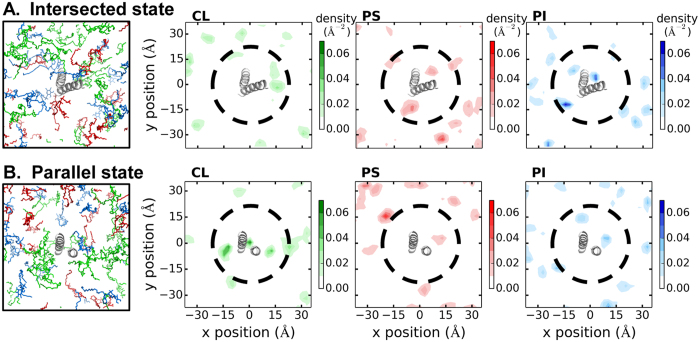
Distributions of three anionic lipids around the (**A**) intersected and (**B**) parallel α9 dimers with the corresponding average headgroup density maps for CL, PS, and PI (at 190–200 ns). The intersected and parallel α9 dimers in the final snapshots (t = 200 ns) are shown in a gray cartoon representation, and anionic lipids are shown in a line representation with CL in green, PS in red, and PI in blue. Black dash circles were drawn at the 20-Å cutoff from the dimer centroids to guide the eyes.

To understand how the membrane stabilizes the α9 dimers, we have investigated the lipid distributions and dimer-lipid interactions. First, we analyzed (Fig. 2) the average anionic lipid distributions and diffusion coefficients in three 10-ns windows during the last 30 ns, and obtained converged results (Supplementary Fig. 4 and Supplementary Table 2) with clear lipid preference near each α9 dimer. Despite the fact that neutral lipids account for ~75% of total lipids in the MOM, the anionic ones such as PS, PI, and CL have been known to facilitate Bax activation, insertion, and pore formation^25^,^26^. Starting with a membrane model that had a random distribution of neutral and anionic lipids, the anionic lipids were enriched around each α9 dimer in the end. An area with a radial cutoff of 20 Å from the dimer centroid contains 24–26% of anionic lipids and 18–21% of neutral lipids on average (given its bulky tails, a CL counts as twice the area of other lipids).

Within the radial cutoff of 20 Å from an α9 dimer centroid, we calculated the stoichiometry of CL, PS, and PI, which is 2.0 ± 0.1, 2.7 ± 0.5, and 2.7 ± 0.5 per intersected dimer, and 3.7 ± 0.5, 1.0 ± 0.1, and 2.0 ± 0.1 per parallel dimer, respectively. Such evident discrepancy suggests a preference of the intersected dimer to PS and PI (that carry one negative charge under the neutral *p*H), in contrast to a preference of the parallel dimer to CL (that carries two negative charges).

**Figure 3:**
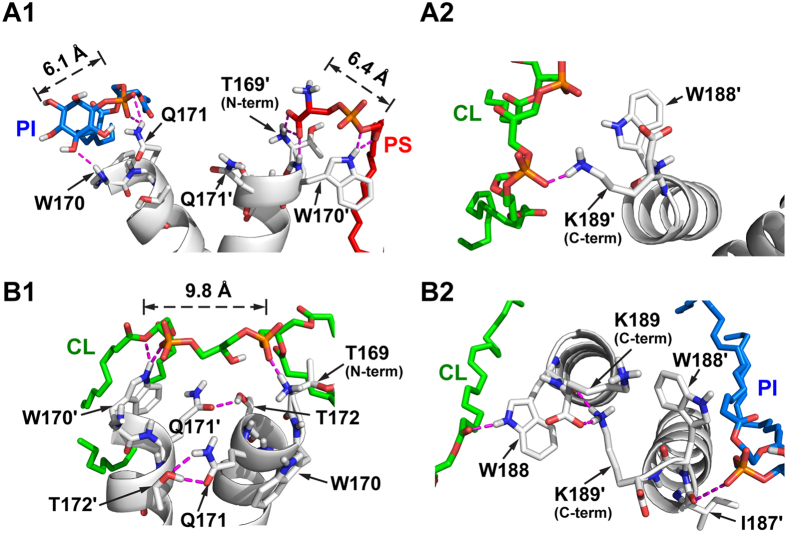
The α9 dimers and their polar interactions with anionic lipids (magenta dashed lines). Nonpolar hydrogens are not displayed. Multiple and frequent contacts: (**A1**) PI-W170, Q171 and PS-T169′, W170′ in the intersected dimer; (**B1**) CL-T169, W170′ in the parallel dimer. Occasional contacts: (**A2**) CL-K189′ in the intersected dimer; (**B2**) PI-I187′ and CL-W188 in the parallel dimer. While few inter-monomer contacts were found in the intersected state near the terminal regions, multiple inter-monomer contacts like Q171-T172′, T172-Q171′, and K189 (COO^−^)-K189′ (εNH^3+^) were observed at the dimer interface in the parallel state (**B1**,**B2**). Rare PS lipids get close enough to the parallel dimer to form polar contacts (Supplementary Figs 5 and 6).

Second, we examined the protein-lipid interacting sites identified from the converged lipid distributions and found a structural basis for the lipid preference. Contacts between the α9 dimer and the headgroups of PS, PI, and CL are found to involve the side chain, backbone, or the terminal groups (–COO^−^ and –NH^3+^) of amino acids (Fig. 3). Around the intersected dimer, PS and PI are able to form multiple polar contacts with each α9 monomer: for example, PI contacts with W170 and Q171, and PS interacts with T169′ and W170′ (Fig. 3A1). However, CL only forms occasional, transient contacts (duration below 1 ns, Supplementary Fig. 5) with residues like K189′ (Fig. 3A2), which is insufficient to keep CL bound to α9. This explains why more PS and PI lipids than CL dwell near the intersected dimer in our simulations. Around the parallel α9 dimer, CL owing to its wide headgroup (~10 Å) is able to span across a large region and form multiple long-lasting contacts with both monomers. However, since more terminal residues participate in the interfacial interaction between two monomers (Fig. 3B1), in the parallel dimer there are fewer residues available for binding PI and PS (Fig. 3B2). As a result, the presence of PI and PS in the vicinity of the parallel dimer is reduced. Therefore, it is the availability of the residues exclusive from the dimer interface and the distinct lipid head-group together that determine the intersected dimer’s preference to PS and PI, and the parallel dimer’s preference to CL.

Our observation, which the α9 dimer in different conformational states preferentially binds anionic lipids, is in line with a previous study showing that the α9-membrane interaction is influenced by the presence of anionic lipids^27^. Further, while previous studies mainly focus on the role of CL and other anionic lipids on the activation of the BH3-only protein Bid or of Bax by a truncated Bid (tBid)^26^,^28^, our study describes a detailed picture of the α9 dimerization that occurs downstream of Bax activation to link the BH3-in-groove Bax dimers into higher-order oligomers. Presumably anionic lipids play manifold roles in such processes. On one hand, they recruit a caspase-cleaved Bid (cBid) to the membrane and then activate tBid that in turn recruits and activates Bax^26^. On the other hand, our findings suggest that α9 forms different dimeric states that interact with selective anionic lipids. Accordingly, Bax traps more anionic lipids into a microdomain via α9 dimers (Supplementary Fig. 5), which may destabilize the membrane by concentrating the negative charges, or by increasing the membrane curvature (as PS generates positive curvature and CL leads to very negative curvature of the membrane^26^). This membrane destabilization likely leads to collapse of the bilayer and formation of proteolipidic pores. Moreover, it is found that the binding affinity of cBid to the membrane without CL is similar to that to the membrane with CL as long as the total negative charge is maintained (e.g. by replacing CL with PS)^26^. However, the tBid conformational changes required for activation of Bax are impaired in the membranes that lack CL. Since the active tBid is enriched in the CL-rich domain where Bax can be recruited, the active Bax may first form the parallel dimer in the CL-rich domain and then convert into the intersected one that retains more PI and PS. In summary, the α9 dimerization may enhance the local Bax concentration to the membrane domains enriched with negatively charged CL, PS and PI, which will eventually stress the membrane and promote proteolipidic pore formation.

#### The helix α9 dimer switches between multiple conformational states in the MOM

**Figure 4:**
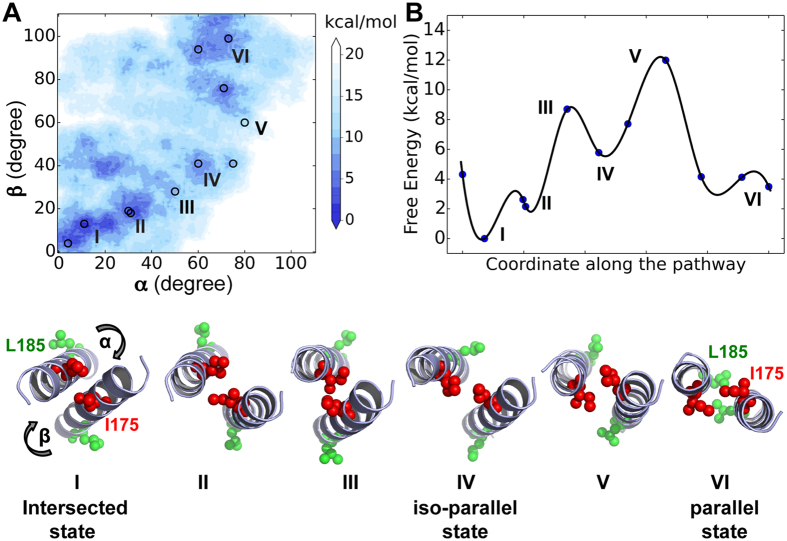
(**A**) The free-energy landscape of α9 dimer transition between the intersected (I) and parallel (VI) conformational states, which are connected by the II–V states. The rotation angles (α, β) of the monomer backbones around the principal helical axes in state I are the collective variables. The free-energy minimum is set to be zero. (**B**) The minimum energy pathway connected by circles in (**A**) with states I and VI labeled and shown in a cartoon representation. Residues I175 and L185 are highlighted by red and green spheres.

According to our conventional MD simulations, the α9 dimers in the intersected and parallel states are stable in the MOM, but interact differently with anionic lipids, and their interconversion is likely related to the Bax activation. To further investigate the conformational transition pathway of the α9 dimer, we employed metadynamics simulations to calculate the free-energy landscape of the α9 dimer in the membrane, regarding two collective variables: the angles of rotation (α, β) of backbone around the principal axis of the two helical monomers. The rotation angles in the starting intersected dimer are set to zero degree. The rotation directions are chosen along the smallest rotation angle to transit. We did not constrain the monomer-monomer separation or the crossing angle, and thus they were allowed to relax when the monomers rotated along the directions shown in Fig. 4. To ensure convergence, we performed two simulations to model the transition: one for the path from the intersected to the parallel dimer while the other for the reverse path. Consistent results have been obtained from these simulations, which confirm the convergence of our free-energy calculations (Supplementary Fig. 7).

**Figure 5:**
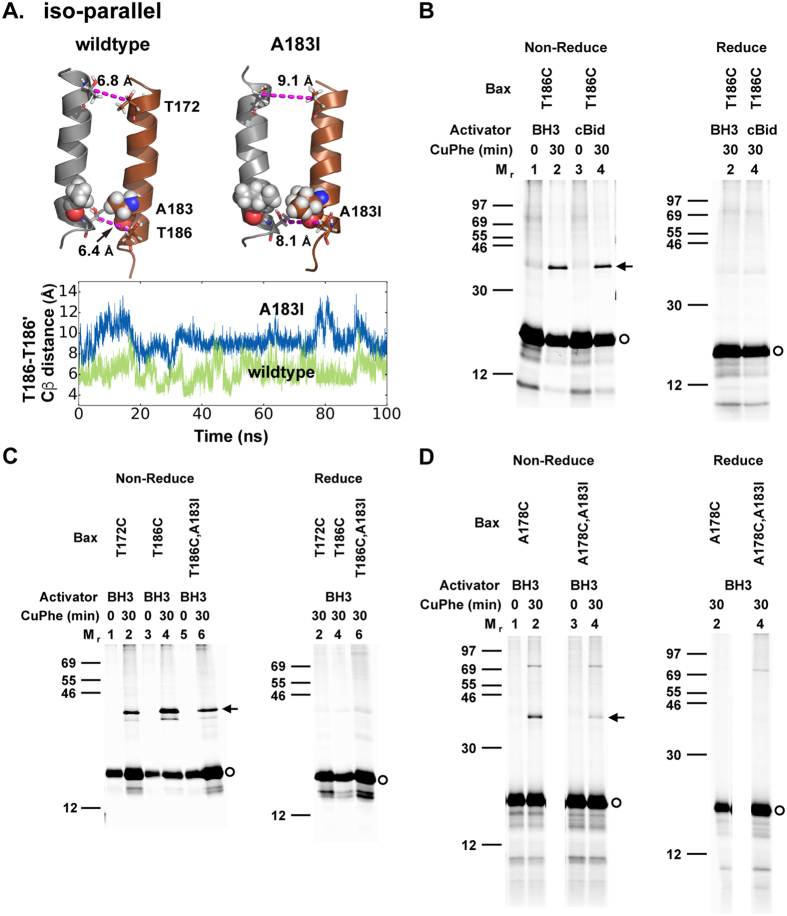
(**A**) Top, the iso-parallel α9 dimer conformation in the absence (left) and presence (right) of the A183I mutation. The C_β_ atoms of the cross-linkable pairs, T172-T172′ and T186-T186′, are linked by dashed line with the distance indicated. Bottom, time evolution of the C_β_ distance of the T186-T186′ pair during MD simulations. (**B**–**D**) Phosphor-images of non-reducing and reducing SDS-PAGE gels show that single-cysteine Bax mutants T172C and T186C formed disulfide-linked homodimer at the mitochondria after activation by either Bax BH3 peptide (BH3) or cBid protein. The A183I mutation inhibited the disulfide crosslinking of the T186C mutant (**C**) and the A178C mutant (**D**). Protein standards are indicated on the left side by their molecular mass (M_r_). Bax monomers and the disulfide-linked dimers are indicated on the right side by circles and arrows, respectively. n ≥ 2.

We then extracted the shortest transit pathway and identified the representative states (Fig. 4 and Supplementary Fig. 8), including four major low-energy states (I, II, IV, and VI) and two high-energy transition states (III and V). States I (α = 0°, β = 0°) and VI (α = ~70°, β = ~90°) correspond to the intersected and parallel dimer states respectively, which fit the crosslinking data from our previous study^12^. Our results suggest that the transition pathway from the intersected conformation toward the parallel one consists of three major steps (Fig. 4B). (i) The α and β rotation angles in the dimer increases to cause a slight increase in the monomer-monomer distance and a decrease in the crossing angle, which enables a transition from state I to II (α = ~30°, β = ~20°). (*ii*) State II converts into state IV (α = ~60°, β = ~40°), where the helix-helix crossing angle gets below 15° and T172 and T186 in one monomer approaches their counterparts in the other monomer at the dimer interface (Fig. 5A, hereafter the state IV is referred to as the *iso-parallel* state). There is an energy barrier of 3.1 kcal/mol (state III) when I175 and I175′ are very close. (*iii*) The monomers in iso-parallel state continue to rotate, resulting in state VI with Q171, A178, T182 and L185 in one monomer closer to their counterparts in the other monomer. Given the transition barrier as high as 6.2 kcal/mol (state V), this step is likely to be rate-limiting in the entire pathway.

Our metadynamics simulations not only are consistent with prior crosslinking data^12^ and conventional MD simulations on the stability of the α9 dimer (Fig. 1C), but also provide valuable quantitative, mechanistic evidence that is not readily available via other approaches. First of all, the free-energy map (Fig. 4A) and the potential transition pathway (Fig. 4B) indicate multiple conformational states of the α9 dimer in the MOM — which is more complex than a simple two-state model. The energy map indicates the conformational diversity of the dimer in the MOM with the intermediate states (including the blue areas not labeled in Fig. 4A) with lower free energies that are more stable than the others with higher free energies. Although the shortest transition pathway between the intersected and parallel states is presented in Fig. 4B, we are also aware of other pathways to connect the multiple dimer states, which can likely promote the conformational heterogeneity. Next, the intersected dimer is found 3.5 kcal/mol lower than the parallel one in free energy. Thus, in the MOM there could be slightly more intersected dimers, since it is thermodynamically more stable. However, the intersected and parallel α9 dimers are likely to coexist, provided an overall barrier of just 12 kcal/mol between them. In addition, we have found the iso-parallel dimer interface as an intermediate state with a small crossing angle (~15 degree) and a moderate monomer-monomer distance. Because of the small energy difference between the iso-parallel (IV) and parallel (VI) states, it is likely that the iso-parallel state is also stable or at least metastable in the membrane. Supportively, the iso-parallel α9 dimer still resembles its initial model from the metadynamics simulation after a 282-ns conventional MD simulation, with a final backbone RMSD as low as 2.2 Å. Therefore, combining our conventional and metadynamics simulations, we are able to support the previous experimental work^12^ and further predict a new dimeric state.

Recently, three compounds targeting at S184 in helix α9 have been suggested as Bax agonists for potential treatments against the lung cancer^29^. The discovery of multiple conformational states proposes the helix α9 dimer as a potential therapeutic target to treat diseases associated with excessive Bax activation or inhibition. To further our understanding towards this goal, we carried out experiments of the helix dimer in intact Bax proteins to validate the iso-parallel state and to show the possibility to perturb selective states.

#### The simulation-predicted iso-parallel α9 dimer was validated in the mitochondria

To determine whether the iso-parallel α9 dimer interface is actually formed by the active Bax proteins in the MOM, we made two single-cysteine Bax mutants T172C and T186C. The cysteine pairs that replace the T172-T172′ and T186-T186′ pairs are within disulfide-linkable distance according to the iso-parallel dimer model (Fig. 5A), but not in the disulfide-linkable distance of the intersected and parallel dimer models. We synthesized the [^35^S]methionine-labeled mutant proteins *in vitro*, activated them by a BH3 peptide from Bax (BH3) or cBid, and targeted them to the mitochondria lacking endogenous Bax and Bak as before^12^. We then oxidized the resultant mitochondria to induce disulfide linkage between each mutant. We used non-reducing SDS-PAGE and phosphor-imaging to detect the radioactive Bax monomer and the potential disulfide-linked dimer. As expected from the iso-parallel α9 dimer model, both single-cysteine Bax mutants formed disulfide-linked homodimers at the mitochondria (Fig. 5B,C). Of note, these *in vitro* synthesized single-cysteine Bax mutants could release cytochrome c from the Bax and Bak deficient mitochondria in a tBid-dependent manner (data not shown). Therefore, the iso-parallel α9 dimer detected by the crosslinking is formed by the active Bax protein.

#### The α9 dimer in different states can be selectively perturbed by mutations

Consistent evidence from both simulations and experiments has shown that the G179I and T182I mutations generate steric clashes in the interfaces to disrupt the intersected and parallel α9 dimers, respectively^12^ (Supplementary Figs 9 and 10). Likewise, as indicated by the simulations, A183I disrupts the iso-parallel α9 dimer and increases the distance of the T186-T186′ pair beyond a cross-linkable range (Fig. 5A).

To verify the simulation-predicted effect of the A183I mutation on the iso-parallel and other α9 dimers, we made the A183I mutation into the T186C or A178C mutant that was used to detect the iso-parallel or parallel dimer in crosslinking experiments, respectively. In accordance to the MD simulations (Fig. 5A), the disulfide crosslinking of the T186C mutant was inhibited by the A183I mutation (Fig. 5C, comparing lane 6 to 4), providing further support to the iso-parallel dimer model. Unexpected from the parallel dimer model but expected from the predicted iso-parallel to parallel transition path, the A183I mutation also inhibited the parallel α9 dimerization detected by the disulfide crosslinking of the A178C mutant (Fig. 5D, comparing lane 4 to 2). Also, we found that the contacts of the iso-parallel dimer to anionic lipids are less than they are around the intersected dimer (Supplementary Fig. 11). Taking the simulation and experimental evidence together, the iso-parallel α9 dimer represents a conformational state formed during the Bax oligomerization, which intermediates the intersected and parallel states, and facilitate their interconversion.

## Discussion

**Figure 6:**
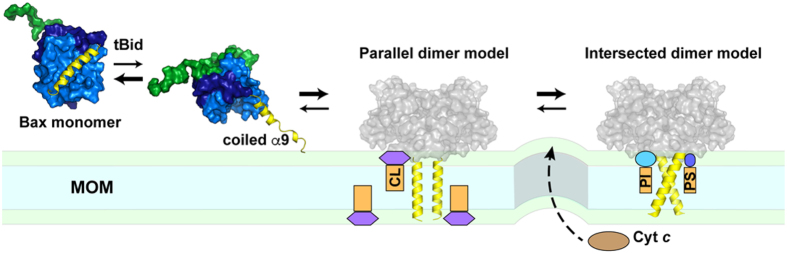
Cartoon illustration of helix α9 in soluble Bax (PDB code: 1F16) and the MOM targeted Bax. The N-terminus and helix α1 are shown as the green surface, while helices α2 to α5 in sky blue and helices α6 to α8 in deep blue, and helix α9 is represented by yellow ribbon. After displaced by tBid from the groove formed by helices α2 to α5, helix α9 loses helicity in the cytosol but then folds back within the MOM. The formation of the intersected and parallel α9 dimers attracts different anionic lipids to the vicinity as indicated, which may increase membrane tension and curvature, thereby promoting proteolipidic pore formation to release cytochrome c.

Connecting the evidence from simulations and experiments, we propose a possible molecular mechanism for the dynamic conformation and interaction of Bax helix α9 to induce apoptotic pores in the MOM (Fig. 6). The cytosolic Bax protein is equilibrated between a fully folded state with the helix α9 buried in the hydrophobic groove (as shown by the NMR structure)^11^ and a partially unfolded state with α9 released from the groove. Interactions with a BH3-only protein such as tBid may shift the equilibrium to the partially unfolded state due to displacement of helix α9 from the hydrophobic groove to an aqueous environment. Such an order-disorder transition drives α9 to the MOM, where α9 folds back to the helical conformation in a disorder-order transition with its hydrophobic core stabilized by the lipid tail region and the polar/charged termini stabilized by the lipid headgroup region.

During the initial activation, Bax is likely targeted to a CL-rich domain in the MOM (usually near the outer-inner membrane contact site) where the activator tBid is likely located^33^,^28^. Through specific interactions, helix α9 traps more anionic lipids that are wandered by. To remain stable in the membrane, helix α9 likely first forms the parallel dimer that preferentially binds CL. Then the parallel dimer relaxes into the intersected dimer that is more stable and able to bind other anionic lipids like PS and PI. The parallel to intersected conformational transition can go through the intermediate iso-parallel state. The dynamics of the α9 dimer conformation may result in the dynamics of anionic lipid distribution, which destabilizes the lipid bilayer to induce a proteolipidic pore that is lined by both Bax oligomers and lipid headgroups, and able to releases cytochrome c and other intermembrane space proteins to initiate apoptosis.

In general, we have combined simulations and experiments to study the detailed conformations and dynamics of the Bax helix α9 dimers in the MOM, a difficult task with any single approach alone. Corroborating evidence is obtained in the conformational stability of the α9 dimer and mutants, the dimer-lipid interacting patterns, and the free-energy landscape to reflect the α9 dynamics — suggesting a detailed mechanism of helix α9 in the Bax-induced membrane poration, which may be facilitated by the interactions between multiple dimer conformational states and anionic lipids in the MOM. Our combined computational/experimental strategy will be useful to further explore the molecular mechanisms of the other membrane embedded/interacting helices in Bax activation and action, or even the mechanisms of other Bcl-2 family members in the MOM.

## Methods and Models

### Models

The intersected and parallel α9 dimer models were generated in our previous study^12^: the intersected model was predicted *ab initio* by CATM program^16^; the parallel model was obtained through a systematic search of the dimer conformational space directed by the disulfide-crosslinking data. These two models fit the disulfide-crosslinking data from the single-cysteine Bax mutants illustrated in Fig. 1.

We used the membrane builder and MD simulator of CHARMM-GUI^30^ to generate the protein-membrane and protein-solvent systems. The lipid bilayer consist of 46.5% L-α-phosphatidylcholine (PC), 28.4% L-α-phosphatidylethanolamine (PE), 8.9% L-α-phosphatidylinositol (PI), 8.9% L-α-phosphatidylserine (PS) and 7.3% cardiolipin (CL) in mole fraction^25^. All systems were solvated with explicit water in the TIP3P model in the periodic box. Counterions Na^+^ and Cl^−^ were added to keep the system charge neutral.

### Simulation setup

Summary of all the MD simulations is provided in Supplementary Table 1. Most simulations have two replicas, of which the longer one lasts 100–200 ns and a shorter one 50–150 ns. The NAMD package^31^ with CHARMM36 force field has been used^32^. Both equilibration and production runs were performed in the NPT ensemble (310 K, 1 bar, Nose-Hoover coupling scheme) with a time step of 2 fs. The particle mesh Ewald (PME) technique was used for the electrostatic calculations. The van der Waals and short-range electrostatics were cut off at 12.0 Å with switch at 10.0 Å. The metadynamics simulations^33^,^34^ were carried out in NAMD to study the transitions between the intersected and parallel α9 dimers, with the assumption that the transition between two conformations is reversible. The sampling bias was applied to two collective variables — the angles of rotation (α, β) of the backbone atoms around the principle axis of each monomeric helix.

### Data analysis

Conformational analyses were performed with VMD^35^ and Pymol (Schrödinger, LLC). Polar contacts within 3.6 Å were shown by Pymol. RMSDs of the dimer and monomer conformations were computed by backbone alignments on their initial models. The points to build the minimum free-energy pathway were extracted every 20 × 20 units area on energy landscape. The lipid headgroup temporal position was used to calculate the lipid distribution and diffusion. We specified the protein in the center of the box with the overall drift of the system removed. In addition, the diffusion coefficient (*D*_L_) of the anionic lipids was calculated from the mean-square displacement (MSD) over time according to equation 1^36^:


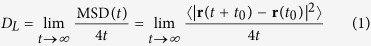


where **r** is the center of mass vector of the lipid headgroup. We calculated the *D*_L_ of each lipid molecule, from which the mean value and standard deviation over all the molecules of the same lipid type were obtained. Both the distribution and the diffusion behavior for each type of anionic lipid were analyzed in three 10-ns windows during the last 30 ns. The comparable *D*_L_ values during these 10-ns windows supports convergence of the lipid diffusion (Supplementary Fig. 4 and Supplementary Table 2).

To determine the existence of the iso-parallel α9 dimer interface, the *in vitro* synthesized [^35^S]Met-labeled single-cysteine Bax proteins, some having the A183I mutation, were activated by either a BH3 peptide from Bax (BH3) or a BH3-only protein (cBid), and targeted to the mitochondria lacking endogenous Bax and Bak. The resulting mitochondria were isolated and oxidized by copper(II) (1,10-phenanthroline)_3_ (CuPhe) for 30 min before adding N-ethylmaleimide (NEM) and EDTA to stop the reaction. For the “0 min” controls, NEM and EDTA were added before adding CuPhe. The resulting samples were analyzed by phosphor-imaging after non-reducing or reducing SDS-PAGE. Representative phosphor-images from two independent experiments were shown in Fig. 5.

## Additional Information

**How to cite this article**: Liao, C. *et al*. Conformational Heterogeneity of Bax Helix 9 Dimer for Apoptotic Pore Formation. *Sci. Rep.* **6**, 29502; doi: 10.1038/srep29502 (2016).

## Supplementary Material

Supplementary Information
