# Supplementary material for: Conformational Heterogeneity of Bax Helix 9 Dimer for Apoptotic Pore Formation
Source: Sci Rep. 2016 Jul 6;6:29502. doi: 10.1038/srep29502 (PMC4933972; doi:10.1038/srep29502)
Supplement: Supplementary Information [file srep29502-s1.pdf]

Supplementary Information

Conformational Heterogeneity of Bax Helix 9 Dimer for Apoptotic Pore Formation

Chenyi Liao[1], Zhi Zhang[2], Justin Kale[3,5], David W. Andrews[3,4,5], Jialing Lin[2,6], Jianing Li[1,*]

[1]Department of Chemistry, University of Vermont, Burlington, VT 05405, USA
[2]Department of Biochemistry and Molecular Biology, and [6]Peggy and Charles Stephenson Cancer Center, University of Oklahoma Health Sciences Center, Oklahoma City, Oklahoma 73126, USA
[3]Biological Sciences, Sunnybrook Research Institute, [4]Departments of Biochemistry and Medical Biophysics, University of Toronto, Toronto, ON, Canada, and [5]Department of Biochemistry, McMaster University, Hamilton, ON, Canada

*Corresponding Author:
Jianing Li (jianing.li@uvm.edu)
Department of Chemistry, University of Vermont, Burlington, VT 05405, USA

Supplementary Table 1. Simulation summary.

Helix α9	Box size (Å^3)	No. of atoms	No. of lipid molecules*	No. of water molecules	Simulation lengths (ns)
Intersected dimer (membrane)	77×77×69	37165	160	4711	100, 200
Parallel dimer (membrane)	77×77×69	37051	160	4673	100, 200
Iso-parallel dimer (membrane)	66×66×64	25767	118	2895	130, 282
Metadynamics simulations	77×77×69	37165	160	4711	30, 30
Intersected dimer (solution)	51×51×51	12264	0	3852	150, 200
Parallel dimer (solution)	55×55×55	15504	0	4930	100, 200
Monomer (solution)	50×50×50	11501	0	3712	85, 60
Intersected G179I dimer (membrane)	77×77×69	37018	160	4654	50, 100
Parallel T182I dimer (membrane)	77×77×69	37088	160	4682	50, 100
Iso-parallel A183I dimer (membrane)	66×66×64	25541	118	2817	120
Total simulation length					2287

* 160 lipids contain 37:23:7:7:6 of PC: PE: PS: PI: CL molecules in each layer; 118 lipids consist of 28:17:5:5:4 of PC: PE: PS: PI: CL molecules in each layer.

Supplementary Table 2. Diffusion coefficients D_L of anionic lipids obtained from the last 30 ns of the 200-ns simulations. Deviations were calculated with all the molecules of the same lipid type.

	**Intersected**			**Parallel**		
D_L (Å^2/ns)	170-180 (ns)	180-190 (ns)	190-200 (ns)	170-180 (ns)	180-190 (ns)	190-200 (ns)
CL	1.6 ± 0.7	1.6 ± 0.6	1.6 ± 0.6	1.4 ± 0.6	1.4 ± 0.5	1.3 ± 0.6
PS	1.7 ± 0.5	1.9 ± 0.6	1.5 ± 0.6	1.6 ± 0.5	2.0 ± 0.9	1.7 ± 0.7
PI	1.3 ± 0.5	1.8 ± 0.7	1.7 ± 0.7	1.6 ± 0.4	1.8 ± 0.6	1.8 ± 0.7

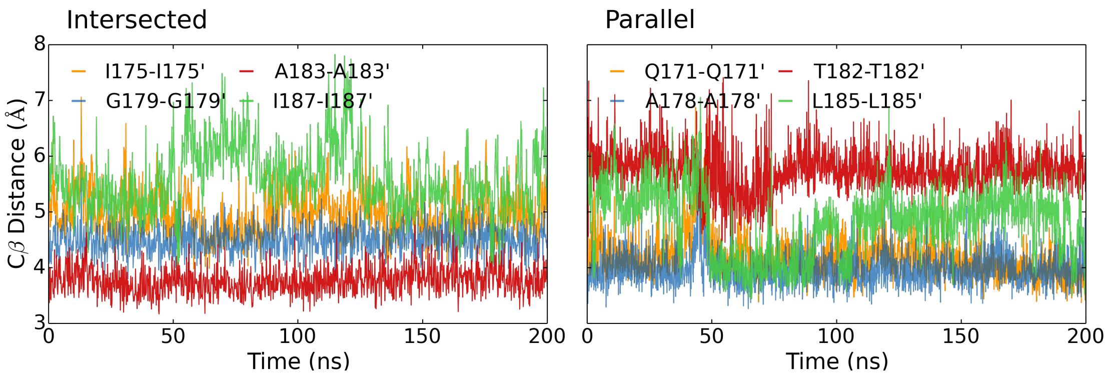

Supplementary Fig. 1. Cβ-Cβ' distance of I175-I175', A183-A183', and I187-I187' pairs in the intersected dimer and Q171-Q171', A178-A178', T182-T182', and L185-L185' pairs in the parallel dimer. For G179-G179' pair in the intersected dimer, Cα-Cα' distance is shown.

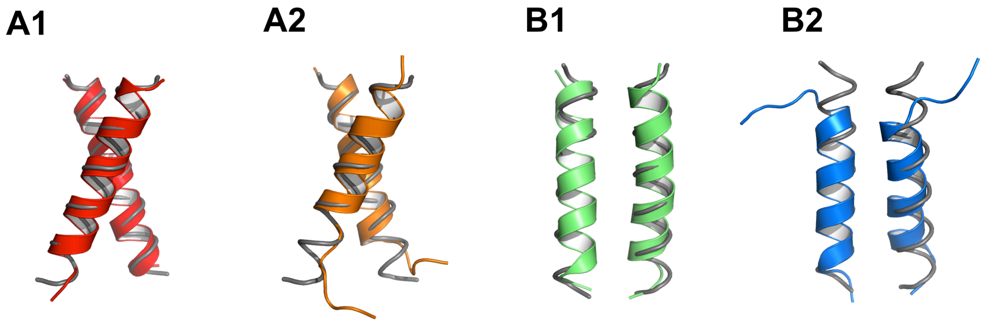

Supplementary Fig. 2. From the second replicas: intersected (A) in different side view from Fig. 1A and parallel (B) helix α9 dimers are stabilized in the membrane (A1, B1) and lost helicity in solution environment (A2, B2). The final dimer conformations in cartoon representations are superimposed to the starting ones as grey tubes.

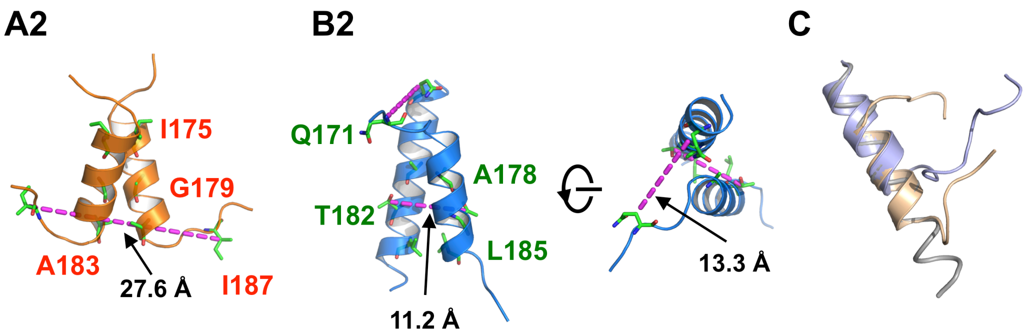

Supplementary Fig. 3. β carbon distance details of (A2) intersected and (B2) parallel α9 dimers in solution. The I187-I187' pair in the intersected dimer dissipated to 27.6 Å, while the Q171-Q171' and T182-T182' pairs in the parallel dimer increase to 13.3 and 11.2 Å, respectively. The dissociated pairs are linked by magenta dashed lines. (C) Final α9 monomers in solution environment from two replicas as light blue and light orange cartoon respectively superimposed to the starting conformation in grey tube.

170-180 ns

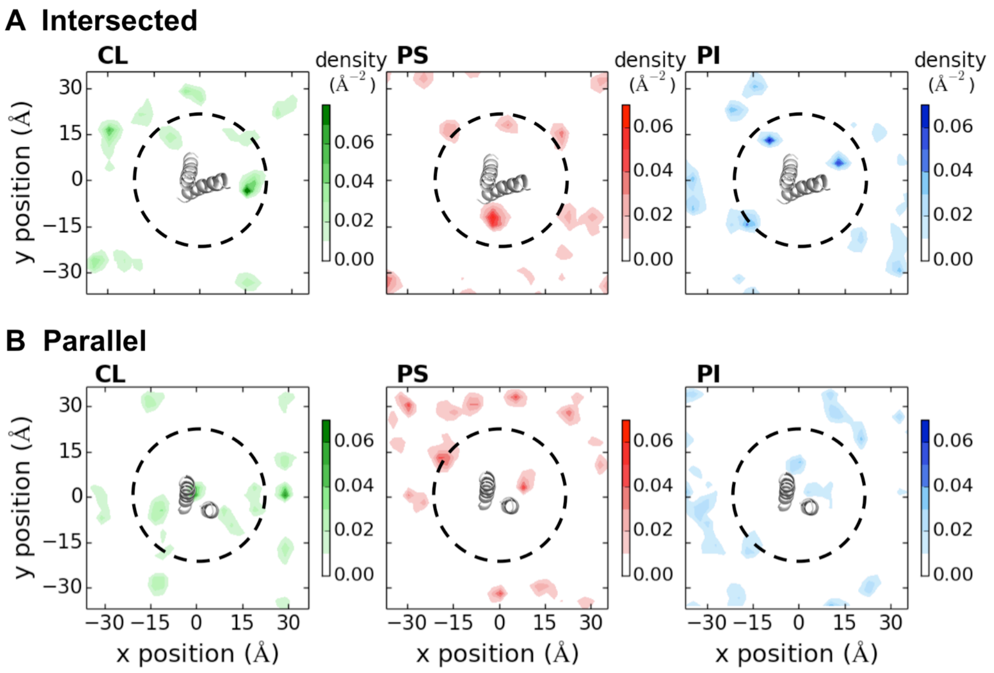

180-190 ns

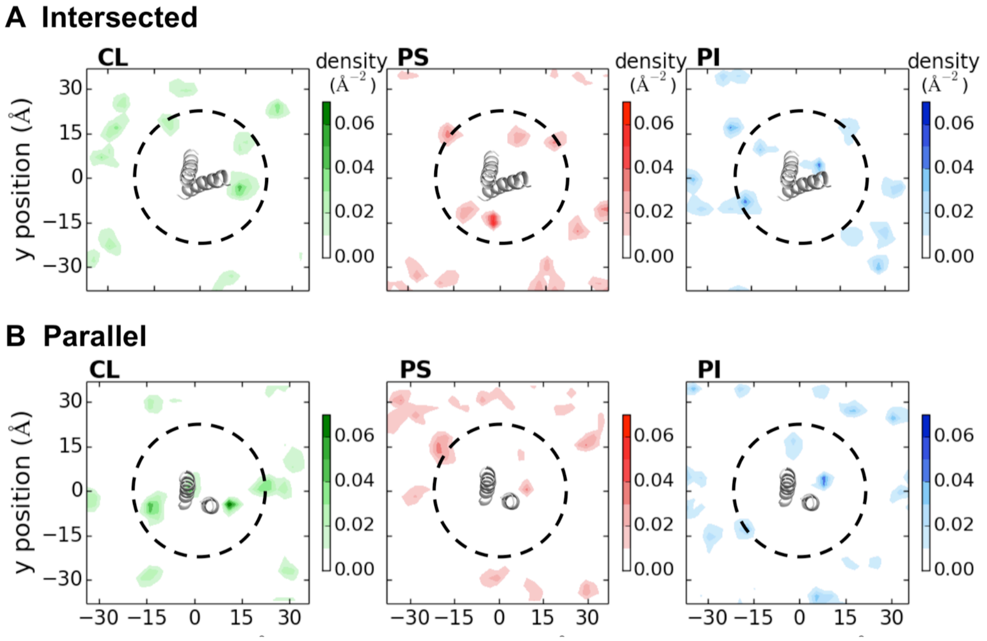

Supplementary Fig. 4. The average headgroup density maps of anionic lipids around (A) intersected and (B) parallel α9 dimers in the periods of 170~180 and 180~190 ns. Anionic lipids are shown as: CL (green), PS (red), and PI (blue). Black dash circles were drawn at the 20-Å cutoff from the dimer centroid to guide the eyes.

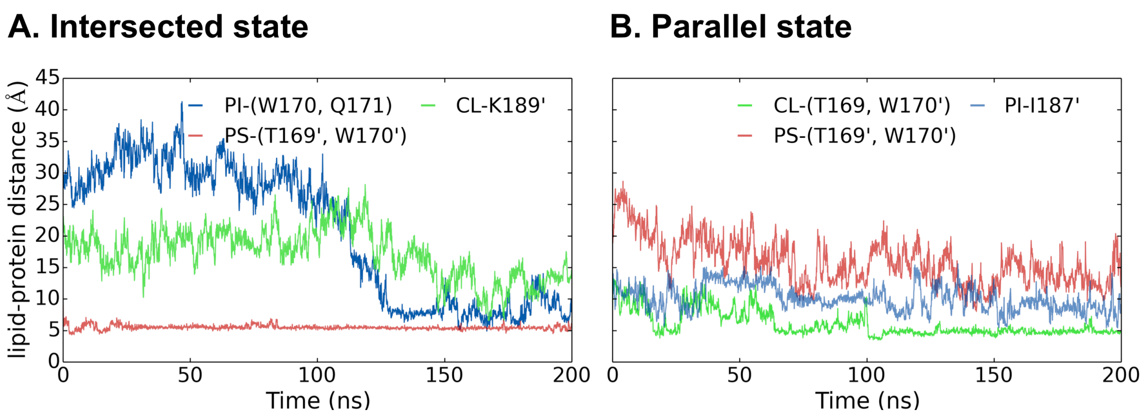

Supplementary Fig. 5. Time evolutions of center of mass distance between the headgroups of anionic lipids and the terminal residues in (A) intersected and (B) parallel α9 dimers corresponding to Fig. 3 and Supplementary Fig. 6. Around the intersected dimer (A), PS (red curve) showed persistent retainability around W170 and Q171 of one monomer. Also, PI (blue curve) became closer to T169' and W170' of another monomer from a very far distance and stayed around. However, CL (green curve) kept in relatively far distance from the intersected dimer with < 1-ns lifetime of the CL-K189 distance within 8 Å. Around the parallel dimer (B), CL (green curve) kept a long-lasting closed distance (~5 Å) with W170 and T169' from both monomers. However, PI (blue curve) only formed transient close distance with the parallel dimer and stay mostly 9 Å away. PS (red curve) stayed even farther away from the parallel dimer (Supplementary Fig. 6)

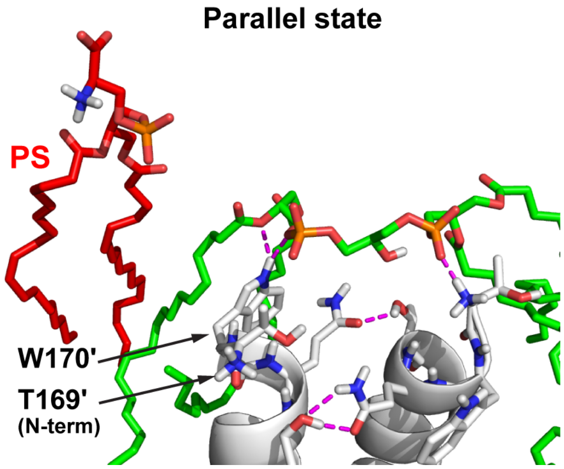

Supplementary Fig. 6. PS and its nearest residues, T169' and W170', of the parallel dimer with same scheme in Fig. 3B1.

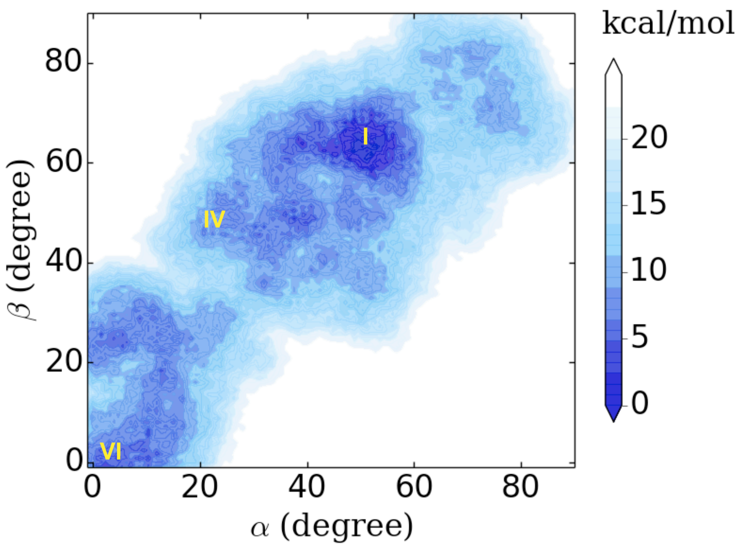

Supplementary Fig. 7. The second replica: free-energy map of a9 dimer transition from parallel state (VI in Fig. 4) to intersected (I in Fig. 4) through intermediate state (IV in Fig. 4). The free energy difference of ~4 kcal/mol between parallel and intersected dimers is consistent with the transition from intersected to parallel transition in Fig. 4A.

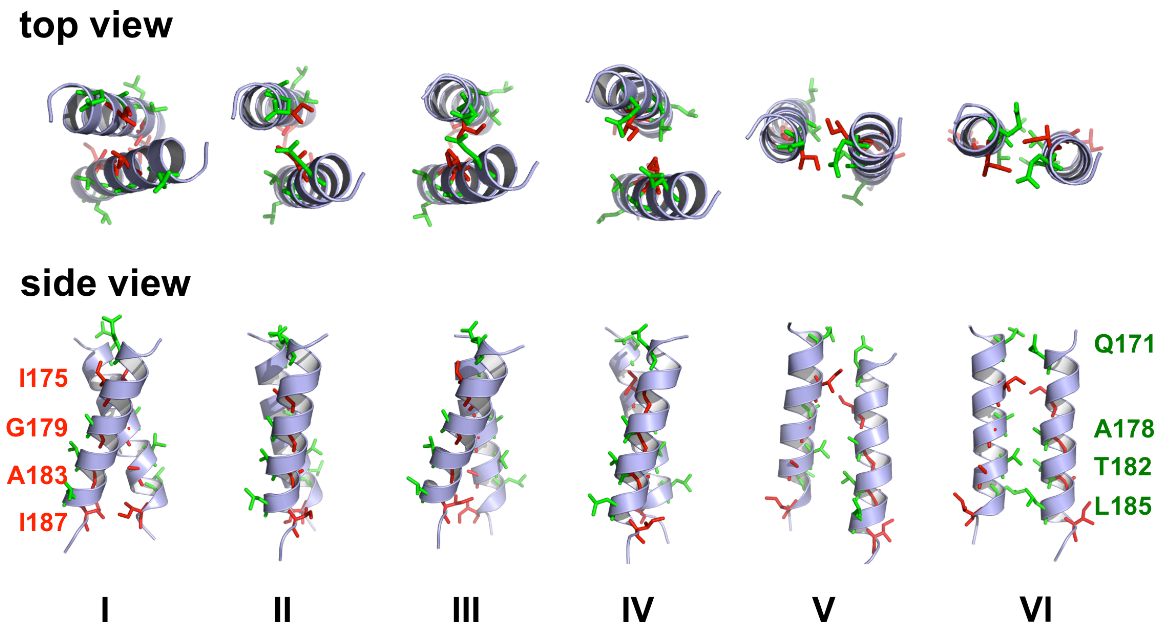

Supplementary Fig. 8. States I ~VI along free-energy path (Fig. 4) in top (upper) and side (bottom) views. Wild-type cross-linkable pairs have been highlighted with stick representations.

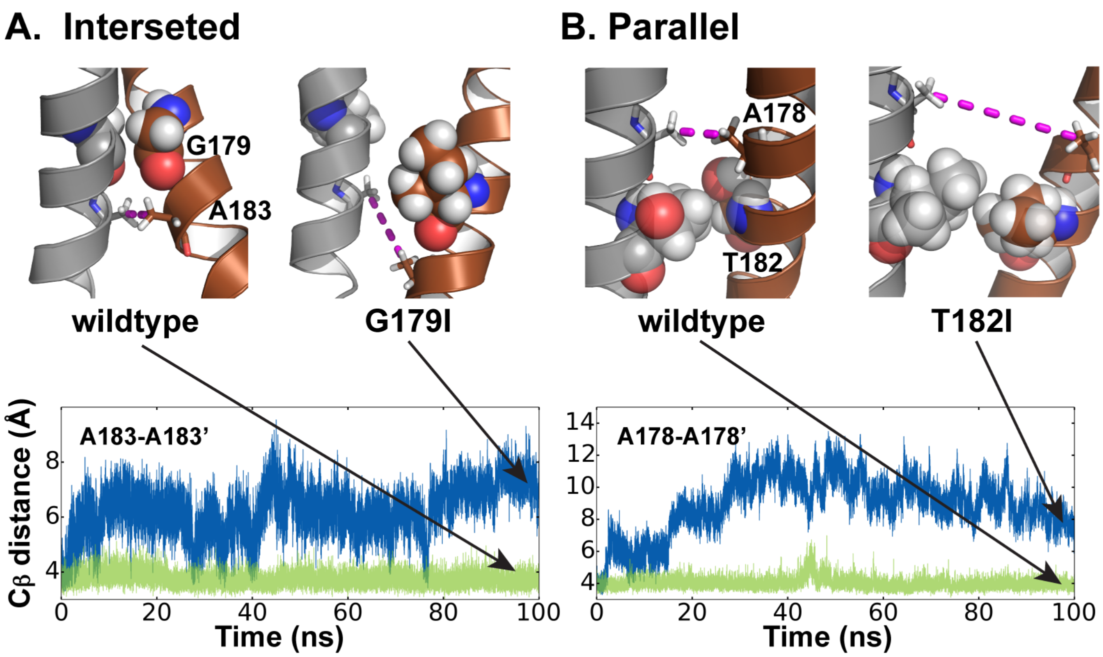

Supplementary Fig. 9. Disruptions of (**A**) intersected and (**B**) parallel dimers by the G179I and T182I mutations as indicated by time evolutions of Cβ distances of the cross-linkable pairs, A183-A183' and A178-A178', respectively. The wild-type and mutated residues 179 in (A) and 182 in (B) are in spheres. The A183 and A183' or A178 and A178' are in sticks connected by a magenta dash through their Cβ atoms. The G179I mutation would generate steric clashes in the tightly packed GxxxA motifs in the intersected a9 dimer. In simulation, the interacted dimer with the G179I mutation shows a forced rotation of the helices as a result of congestion. At the end, the Cβ distance between A183 and A183' increases to near 8 Å (see A). Thus the two residues will not be crosslinked by a disulfide when they are replaced by cysteines, providing an explanation to our experimental observation (Zhang *et al.*[1] Fig. 7B, compare lane 4 to 2 to see G179I inhibition of Bax A183C crosslinking in mitochondria). In the parallel a9 dimer, the T182I mutation would not only produce minor steric clashes in the interface but also change from polar residue to hydrophobic one that reduces the intramolecular backbone-side chain hydrogen bonding. This mutation gradually increase the Cβ distance between the residues A178 and A178' to over 10 Å in simulation (see B), consistent with our observation that this mutation inhibited the disulfide crosslinking of Bax A178C mutant in mitochondria (Zhang *et al.*[1] Fig. 7B, compare lane 8 to 6).

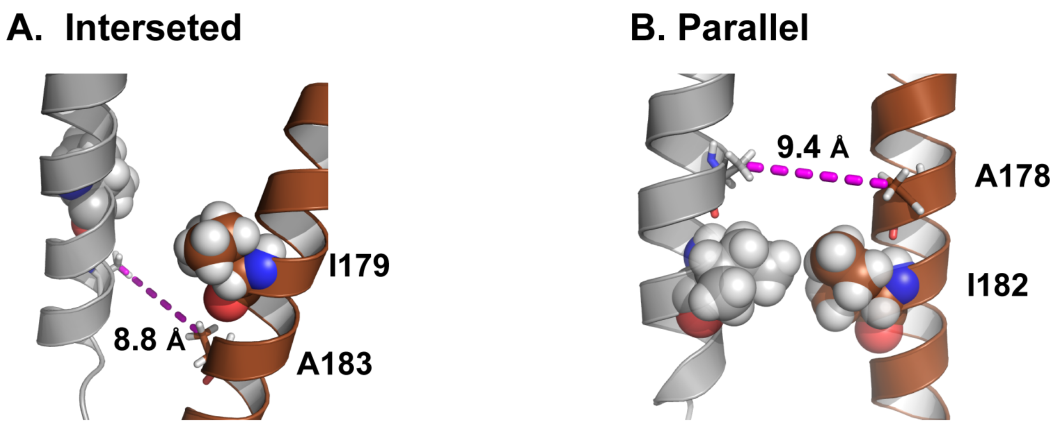

Supplementary Fig. 10. From the second replicas: final snapshots of G179I and T182I mutants in intersected (A) and parallel (B) α9 dimers in membrane at 50 ns in replica. Mutated residues, I179 and I182 are in sphere. The wild-type cross-linkable pairs A183-A183' and A178-A178' (in stick) are connected in dash. Results are qualitatively consistent with Supplementary Fig. 9.

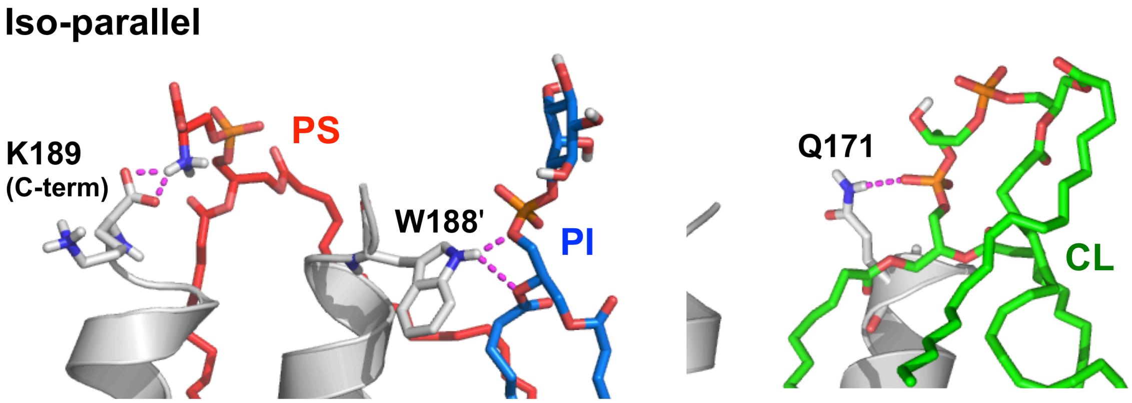

Supplementary Fig. 11. The iso-parallel α9 dimer and its polar interactions with anionic lipids (magenta dashes). PS and PI only form double contacts with K189 and W188', which are less than them are in the intersected dimer (Fig. 3A1).

Reference

1 Zhang, Z. *et al.* BH3-in-groove dimerization initiates and helix 9 dimerization expands Bax pore assembly in membranes. *EMBO J.* **35**, 208-236 (2016).
